# River Boats Contribute to the Regional Spread of the Dengue Vector *Aedes aegypti* in the Peruvian Amazon

**DOI:** 10.1371/journal.pntd.0003648

**Published:** 2015-04-10

**Authors:** Sarah Anne Guagliardo, Amy C. Morrison, Jose Luis Barboza, Edwin Requena, Helvio Astete, Gonzalo Vazquez-Prokopec, Uriel Kitron

**Affiliations:** 1 Department of Environmental Sciences, Emory University, Atlanta, Georgia, United States of America; 2 Department of Entomology, University of California, Davis, Davis, California, United States of America; 3 Universidad Nacional de la Amazonía Peruana, Iquitos, Peru; 4 U. S. Naval Medical Research Unit No.6 (NAMRU-6) Iquitos Laboratory, Iquitos, Peru; 5 Fogarty International Center, National Institutes of Health, Bethesda, Maryland, United States of America; Centers for Disease Control and Prevention, Puerto Rico, UNITED STATES

## Abstract

**Background and Objectives:**

The dramatic range expansion of the dengue vector *Aedes aegypti* is associated with various anthropogenic transport activities, but little is known about the underlying mechanisms driving this geographic expansion. We longitudinally characterized infestation of different vehicle types (cars, boats, etc.) to estimate the frequency and intensity of mosquito introductions into novel locations (propagule pressure).

**Methods:**

Exhaustive adult and immature *Ae*. *aegypti* collections were performed on six different vehicle types at five ports and two bus/ taxi departure points in the Amazonian city of Iquitos, Peru during 2013. Aquatic vehicles included 32 large and 33 medium-sized barges, 53 water taxis, and 41 speed boats. Terrestrial vehicles sampled included 40 buses and 30 taxis traveling on the only highway in the region. *Ae*. *aegypti* adult infestation rates and immature indices were analyzed by vehicle type, location within vehicles, and sampling date.

**Results:**

Large barges (71.9% infested) and medium barges (39.4% infested) accounted for most of the infestations. Notably, buses had an overall infestation rate of 12.5%. On large barges, the greatest number of *Ae*. *aegypti* adults were found in October, whereas most immatures were found in February followed by October. The vast majority of larvae (85.9%) and pupae (76.7%) collected in large barges were produced in puddles formed in cargo holds.

**Conclusions:**

Because larges barges provide suitable mosquito habitats (due to dark, damp cargo storage spaces and ample oviposition sites), we conclude that they likely serve as significant contributors to mosquitoes’ propagule pressure across long distances throughout the Peruvian Amazon. This information can help anticipate vector population mixing and future range expansions of dengue and other viruses transmitted by *Ae*. *aegypti*.

## Author Summary

The dengue vector, *Aedes aegypti*, is an invasive mosquito that is currently in the process of expanding geographically from urban to peri-urban and rural sites throughout Latin America. To improve our understanding of *Ae*. *aegypti* population mixing and how it is introduced to new areas, we investigated the infestation levels of different aquatic and terrestrial vehicles in the Peruvian Amazon. Our results show that large barges are heavily infested with *Ae*. *aegypti*, and therefore are likely to play an important role in *Ae*. *aegypti* geographic expansion.

## Introduction

Anthropogenic changes such as increased trade, rapid transportation, and population movements favor the introduction and establishment of invasive mosquitoes and pathogens [1]. Recent reports of dengue in Key West, Florida [2], West Nile Virus in the United States [3], and Chikungunya in the Caribbean [4] demonstrate the potential for introduced mosquitoes and pathogens to cause serious outbreaks. Accordingly, an understanding of the biological and ecological factors that facilitate establishment of a vector species in a new location can provide timely information to develop and implement vector surveillance and control, and in particular to suppress the geographic expansion of vector-borne pathogens.

An invasion process can be summarized as a series of sequential steps including transport to a new region, release into the new environment, establishment, and spread [5]. Several factors determine the success of an invading organism, including the number and frequency of introduction events (propagule pressure), key life history traits, behavior of the invading species, and abiotic and biotic properties of the receiving ecosystem [5–8]. Dengue vector *Aedes aegypti* displays several characteristics that contribute to its rapid and ongoing spread through human transport activities including; egg desiccation resistance, anthropophilic blood-feeding, and oviposition in artificial water-holding containers commonly found in and around the home such as vases, plastic buckets, water storage tanks, and discarded refuse and tires [9–11].

*Ae*. *aegypti* dispersal occurs in one of two ways: 1) Adult *Ae*. *aegypti* may fly when seeking human hosts or oviposition sites, and 2) *Ae*. *aegypti* eggs, larvae, pupae, or adults may be passively transported from one place to another via anthropogenic activities. It is generally accepted that *Ae*. *aegypti* flight range is limited to ~100m and often much less [12–14], and therefore human activities are responsible for mosquito dispersal over longer distances. Human-mediated dispersal is supported by evidence from population genetics studies [15–17], in addition to field studies documenting *Ae*. *aegypti* on airplanes, boats, and trains [9,18,19]. Indeed, *Ae*. *aegypti* most likely was transported from West Africa to the Americas via trade ships in the 15^th^-19th centuries [10,20].

Since the waning of a Pan American Health Organization yellow fever control program in the 1960–70s [21,22], *Ae*. *aegypti* has been expanding from urban to peri-urban and rural areas throughout the Americas, including the Peruvian Amazon [23,24]. Our previous research demonstrates different spatial patterns of *Ae*. *aegypti* infestation in communities accessible by roads vs. rivers. *Ae*. *aegypti* expansion follows the linear configuration of highway communities, whereas no clear pattern exists in riverine communities [23]. Although environmental differences between settlements (i.e.—abundance of wet containers) may contribute to the heterogeneous infestation pattern, it is also possible that varying degrees of frequency and intensity of new mosquito introductions (propagule pressure) contribute to the spatial pattern of *Ae*. *aegypti* geographical spread. That is, some towns are likely to have more frequent introductions than others.

Insect vector invasion via human transportation networks has been previously described, e.g., *Culex quinquifasciatus* transport via airplanes in the Galapagos [25], *Triatoma infestans* movement via human activities in Peru [26], and *Aedes albopictus* movement through tire trade [27]. Morrison et al (2006) showed *Ae*. *aegypti* infestation in Iquitos ports and in large barges, but did not characterize temporal trends of infestation or the extent of vehicle infestation patterns [18]. In the present study we specifically address propagule pressure through an analysis of *Ae*. *aegypti* infestation rates of different vehicle types in a relatively isolated region of the Peruvian Amazon. We compared *Ae*. *aegypti* adult and immature infestation levels of various vehicle type (boats, buses, taxis, etc.) and between periods of high and low precipitation. Our approach provides empirical data on vehicle infestation that can be combined with transportation data to provide more accurate estimates of propagule pressure, thus aiding in our ability to ultimately predict *Ae*. *aegypti* range expansion and mitigate future dengue outbreaks.

## Methods

### Ethics Statement

Permission for this study was granted by the Loreto Regional Health Department, and the study protocol was approved by the NAMRU-6 Institutional Review Board in compliance with all applicable Federal regulations governing the protection of human subjects (protocol number NAMRU6.2012.0039). Vehicle operators provided oral consent to the collection of mosquitoes, and no personal information was collected during entomological surveys. In accordance with the NAMRU-6 IRB-approved protocol, we provided vehicle operators with handouts documenting mosquito collection procedures in detail. Written consent was not appropriate for this study, since our project only involved the collection of mosquitoes and no personal information was collected. In addition, the Emory University Institutional Review Board determined that this study does not represent human subjects based research.

### Study Area

Iquitos is the most populous city in the Peruvian Amazon, with approximately 400,000 inhabitants in the metropolitan area. Although river networks are the predominant mode of transit, a 95 km road connecting Iquitos to the smaller city of Nauta (population: 17,000), facilitates terrestrial commerce and population movement. Seasonal fluctuations in Amazon River levels influence the degree of transit within the Peruvian Amazon: river transit is most intense when the river levels are intermediate (September-January, April-June) and less frequent during periods of extreme high or low river levels (highest in ~March, lowest in ~August) [28].

### Entomological Sampling

In February, May, August, and October of 2013 we surveyed different vehicle types for *Ae*. *aegypti* adult and immature mosquitoes. Weather in Iquitos exhibits seasonality, but the magnitude of change in temperature and precipitation is small [28]. Although precipitation occurs throughout the year, it is usually lowest between May and September (**S1 Fig**). Thus, we carried out two collections during periods when precipitation was high (February and October) and two collections during periods when precipitation and temperatures were lowest (May and August). The high precipitation period coincides with the dengue season in Iquitos, occurring from September to April [28].

**Fig 1:**
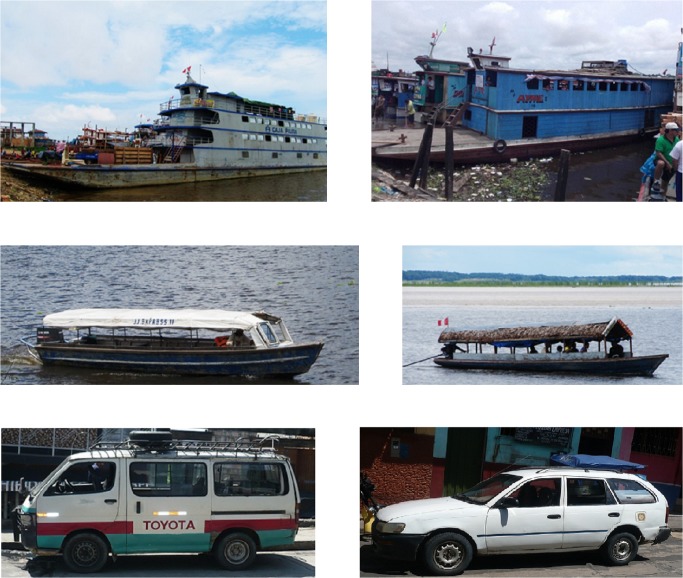
Vehicle types surveyed. Aquatic vehicles surveyed included (clockwise from upper left); large barges (*lanchas*), medium-sized barges (*lanchitas*), small water taxis (*peque-peques*), and speed boats (*rápidos*). Terrestrial vehicles surveyed included buses (*combis*) and taxis.

**Fig 2:**
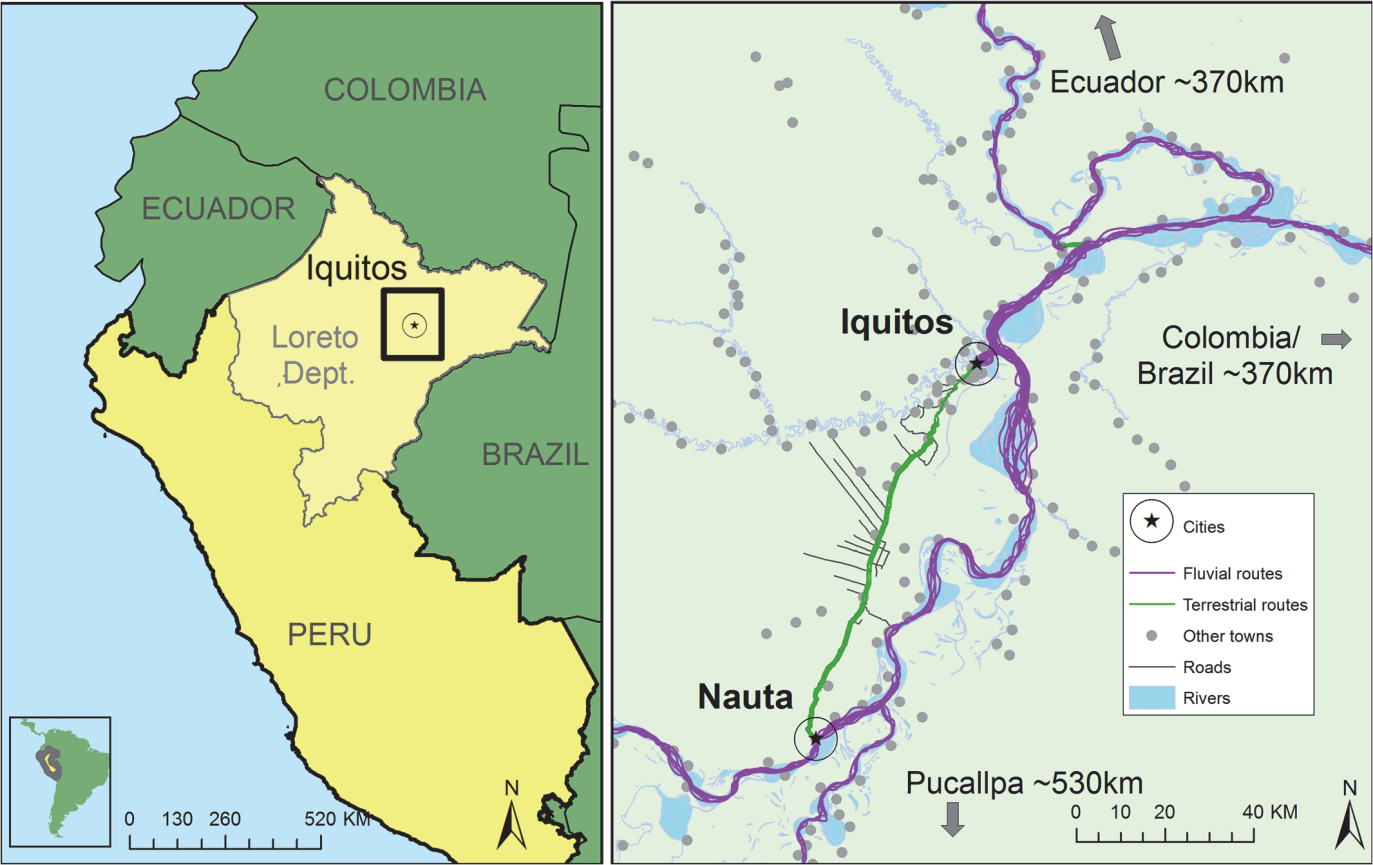
Common transportation routes in the Iquitos region. Transportation is dominated by fluvial activity, with the exception of a 95km highway running from Iquitos to Nauta.

Six different aquatic vehicle types and two different terrestrial vehicle types were surveyed. Aquatic vehicles included large barges (locally known as *lanchas*), medium-sized barges (*lanchitas*), speed boats (*rápidos*), and small water taxis (*peque*-*peques*) (**Fig 1**). Large barges (length ~30m) carry passengers and cargo throughout the Peruvian Amazon and have 2–3 floors including cargo holds in the bottom of the boat. Medium-sized barges (length ~20m) carry cargo and passengers locally in the Iquitos region and have 1–2 floors, but no cargo holds. Terrestrial vehicles included van-sized buses (*combis*) and taxis that travel along the Iquitos-Nauta highway (**Fig 2**), the only major road out of Iquitos, a city which is only accessible by airplane or river travel (minimum of 3 days). For each sampling period, we surveyed between 7 and 17 vehicles of each type for adult and larval mosquitoes.

We surveyed boats and taxis/ buses at five different ports and three different bus stations/ taxi departure points in Iquitos. Vehicles were selected on the basis of departure times (only vehicles staying in port less than two days were included) and the willingness of the owners to participate in the study. We recorded the vehicle name and registration number (when applicable) to uniquely identify each vehicle.

Adult mosquitoes were captured using Prokopack aspirators [29] along the walls of each vehicle, and on every floor including cargo holds. Collection effort was proportional to vehicle size. All adult mosquitoes were transported to the lab, killed by freezing at -20°C, and identified to species using taxonomic keys. When identification to species was not possible due to damage or descaling, mosquitoes were identified and tallied by genus. Male and female mosquitoes were separated and counted, and physiological stage for females was evaluated according to three categories: empty, partially engorged, or completely engorged. When applicable, we also recorded the locations of the mosquito collections within the vehicles (floors 1–3 or cargo holds).

We searched all vehicles for immature mosquitoes, although they were only found on large and medium barges. We thoroughly searched every floor of barges including cargo holds for immature mosquitoes in water-holding (wet) containers. In accordance with previously established protocols [11,30], for each wet container we recorded the location by floor and room type (i.e.- cargo hold, kitchen, etc.), degree of organic material in the water (on a scale of 1 to 3, with 3 representing high concentrations of organic material), container type, solar exposure, fill method (active collection of water via human activities vs. passive, unintentional accumulation of water), presence of abate, and the presence of other mosquito species. All immature mosquitoes were collected in Whirl-pack bags (Nasco, Fort Atkinson, WI) and transported to the laboratory for rearing and identification to species using taxonomic keys. Immature mosquitoes were counted and tallied by species and life stage (egg, larvae, or pupae). After collecting immature mosquitoes, we either emptied the wet containers or we treated the container with Abate (temephos) larvicide to prevent further proliferation of mosquito populations.

### Transportation Data

Simultaneous with entomological surveys, we interviewed vehicle drivers to determine the frequency of travel between Iquitos and surrounding towns for each vehicle type (and thus we were able to estimate the number of vehicles traveling to each town). Additionally, the final destinations for each vehicle were mapped using ArcGIS, and the path distances between final destinations and Iquitos were measured. We then calculated the average distance traveled for each vehicle type infested with *Ae*. *aegypti* mosquitoes.

### Data Management and Analysis

Due to unpredictable vehicle transit patterns in Iquitos, on a number of occasions we sampled the same barge more than once. In order to adhere to statistical assumptions of independence we randomly selected which observation would be included in the dataset for all statistical tests. We assigned a unique identifier to each individual vehicle, and all vehicles that were only sampled on one occasion were included in the final subset of data. For vehicles that were sampled on multiple occasions, we assigned an additional identifier representing the sampling occasion. (For example a vehicle surveyed in both February and May would be assigned a sampling occasion numbers 1 and 2, respectively.) The sampling instance to be included in the subset of data was randomly selected using a random number generator. Subsampling left us with a total of 32 independent collections from large barges, 33 medium barges, 41 speed boats, 53 water taxis, 40 buses, and 30 taxis.

Fisher’s exact tests were used to determine whether there was variation in proportion of infested vehicles within and between sampling periods. Further analysis was conducted only for large and medium-sized barges, where the vast majority of *Ae*. *aegypti* and other mosquitoes were found.

For these large and medium barges, the number of *Ae*. *aegypti* adults (total, females, and blood-feds) and immatures (larvae and pupae) per barge was calculated by date. Entomological indices were calculated, included the Premise (vehicle) Index (positive vehicles/ number inspected *100), the Container Index (positive containers/ number inspected *100), and the Breteau Index (positive containers/ vehicles inspected*100) [18]. Pupal productivity was calculated by location within the boat (by floor) and by wet container type [11]. Differences in abundance by location within medium barges were not tested, due to overall low *Ae*. *aegypti* abundance. Fisher’s exact tests were used to compare the proportion of infested vs. uninfested containers by container type.

Nonparametric Kruskal-Wallis tests for median comparisons among two or more groups were used to test the null hypothesis of no significant differences in *Ae*. *aegypti* abundance (for either adult or immature mosquitoes) by date or by location within boats (floors 1–3, cargo holds). Abundance comparisons between the high precipitation (February, October) and low precipitation (May, August) dates were made using non-paired Mann-Whitney Wilcoxon tests. We also used non-paired Mann-Whitney Wilcoxon tests to determine whether adult and immature *Ae*. *aegypti* abundance was greater for Puerto Masusa (Iquitos’ largest port) in comparison with all other sampling locations.

All data analysis was conducted using R Statistical Software [31].

## Results

**Fig 3:**
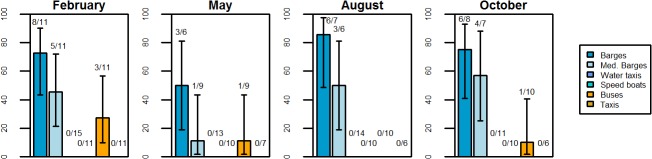
Proportion of vehicles infested with *Ae*. *aegypti* mosquitoes. Vehicles were considered to be infested with *Ae*. *aegypti* if either adult or immature mosquitoes were found. Bars show 95% confidence intervals for proportions. Note that some barges were sampled repeatedly across seasons (N = 14), and within seasons (N = 2).

For all dates, large barges were the most heavily infested with *Ae*. *aegypti*, with an overall infestation rate of 71.9%, followed by medium barges (39.4% infested) and buses (12.5% infested) (**Fig 3**).No *Ae*. *aegypti* mosquitoes were found on taxis, water taxis, or speed boats. Differences in the proportion of vehicles infested were statistically significant within all months except for May (Fisher’s exact test p< 0.003 in all other cases). Since the majority of mosquitoes were found on large and medium-sized barges, the remainder of our analysis is focused exclusively on those vehicle types. **S1–S5 Tables** show a complete list of all mosquito species found by vehicle type.

Large barges traveled extensively throughout the Peruvian Amazon, with an average path distance of 570 km per trip, conducting 3.2 trips per month. Medium barges traveled an approximate path distance averaging 195 km from Iquitos, with 6.5 trips per month (approximately 1/3 of the total distance of large barges per trip, with twice as many trips each month). Buses traveled more locally but with much greater frequency, with a path distance of ~76 km, and an average of 51 trips per month.

### Large Barges

A total of 9 species of mosquitoes from six different genera (all of which have been previously identified in the Iquitos region) were found on large barges (**S1 Table**) [32,33]. Of the mosquitoes identified to species (N = 3850), *Culex quinquefaciastus* was the most common (62.6%), followed by *Ae*. *aegypti* (29.8%), and *Culex coronator* (2.5%) (**Table 1**, **S1 Table**). *Cx*. *quinquefaciastus* and *Ae*. *aegypti* remained the two dominant mosquito species throughout all sampling dates. Of the 32 individual barges sampled, adult *Ae*. *aegypti* were found in 23 barges, and immature *Ae*. *aegypti* were found in 7 large barges. Among large barges that were positive for immature *Ae*. *aegypti*, 6 out of 7 also contained adults.

**Table 1:** Most commonly found adult mosquitoes found on large and medium barges and buses.

	*Culex* (*Culex*) *quinquefasciatus*			*Culex* (*Culex*) *coronator*			*Aedes* (*Stegomyia*) *aegypti*		
	Large Barges	Med. Barges	Buses	Large Barges	Med. Barges	Buses	Large Barges	Med. Barges	Buses
**All Months**	2409	1060	4	96	8	0	1110	79	7
**February**	697	9	1	95	8	0	89	10	3
**May**	510	579	2	1	0	0	144	7	3
**August**	219	65	0	0	0	0	52	9	0
**October**	983	407	1	0	0	0	825	53	1

The most common mosquito species found included *Cx*. *quiquefasciatus*, *Cx*. *coronator*, and *Ae*. *aegypti*. (See S1–S5 Tables Tables for a complete list of mosquito species found by vehicle type.)

**Fig 4:**
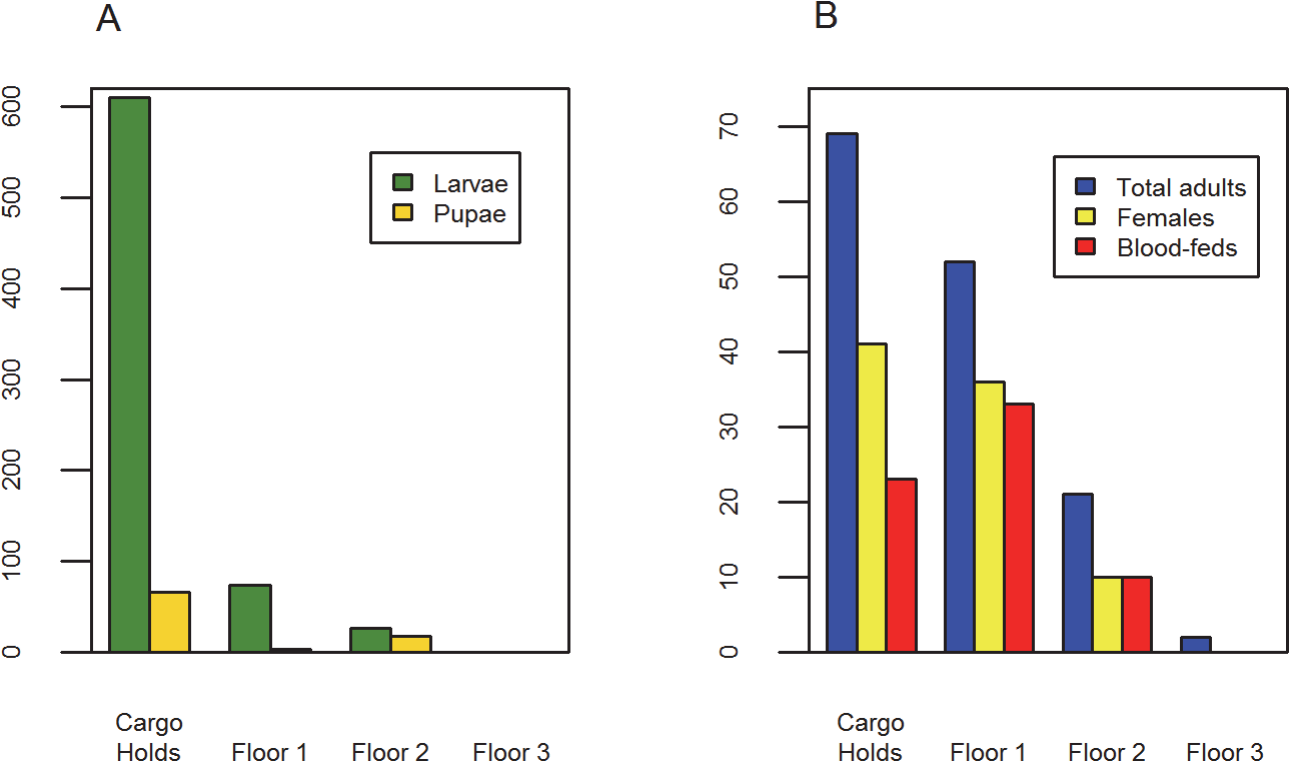
*Ae*. *aegypti* adults and immatures per boat by location for all periods—large barges. The vast majority of *Ae*. *aegypti* immature mosquitoes (A) and adult mosquitoes (B) were found in cargo holds.

*Ae*. *aegypti* adult abundance was highly aggregated—approximately 25% of large barges (N = 8) were responsible for 77.8% of all *Ae*. *aegypti* adults found. Adult *Ae*. *aeygpti* abundance did not differ significantly by date or between high and low precipitation periods. Approximately 75% of female *Ae*. *aegypti* collected (N = 66) were blood-fed (**Table 2**). Within boats, cargo holds had a higher average number of adult *Ae*. *aegypti* than floors 1–3 (Kruskal-Wallis Χ^2^ = 9.80, p< 0.05) (**Fig 4**). This pattern was consistent across all dates, except for August when overall abundance was low and slightly more mosquitoes were found on the first floor (1.6 mosquitoes/ boat on the first floor vs. 1 mosquito/ boat in the cargo holds). Adult *Ae*. *aegypti* were more likely to be found in large barges in Iquitos’ busiest port, Puerto Masusa, in comparison with all other ports sampled (Mann-Whitney Wilcoxon U = 182.5, p<0.05).

**Table 2:** *Ae*. *aegypti* adult mosquitoes on large and medium barges.

	February		May		August		October		All Months	
	Large Barges	Med. Barges	Large Barges	Med. Barges	Large Barges	Med. Barges	Large Barges	Med. Barges	Large Barges	Med. Barges
**No. Barges Sampled**	11	11	6	9	7	6	8	7	**32**	33
**No. Adults (Adults/ Barge)**	58 (5.27)	8 (0.73)	14 (2.33)	1 (0.11)	22 (3.14)	6 (1)	50 (6.25)	8 (1.14)	**144 (4.50)**	**23 (0.70)**
**No. Females (Females/ Barge)**	35 (3.18)	3 (0.27)	5 (0.833)	0	16 (2.29)	4 (0.67)	31 (3.88)	7 (1)	**87 (2.72)**	**14 (0.42)**
**No. Blood-feds (Blood-feds/ Barge)**	17 (1.56)	1 (0.09)	4 (0.67)	0	15 (2.14)	4 (0.67)	30 (3.75)	6 (0.86)	**66 (2.06)**	**11 (0.33)**

The data shown in the table below only includes independent instances of sampling for large (N = 32) and medium (N = 33) barges. Numbers in parenthesis refer to the proportion of adult mosquitoes, females, or blood-fed mosquitoes per barge.

*Cx*. *quinquefasciatus* and *Ae*. *aegypti* were the only two species of immature mosquitoes found in large barges. No differences in the numbers of *Ae*. *aegypti* pupae or larvae were detected by sampling date or by low vs. high precipitation months (**Table 3**). The proportion of positive containers was highest in October (Container Index: 9.8%), and the number of positive containers per 100 vehicles (Breteau Index: 85.7) was highest in August.

**Table 3:** Immature indices by month for large and medium barges.

	February		May		August		October		All Months	
	Large Barges	Med. Barges	Large Barges	Med. Barges	Large Barges	Med. Barges	Large Barges	Med. Barges	Large Barges	Med. Barges
**No. barges sampled**	11	11	6	9	7	6	8	7	**32**	**33**
**Positive Barges**	1	1	0	0	4	1	2	0	**7**	**2**
**Premise Index**	9.09	9.09	0	0	57.14	16.67	25	0	**21.88**	**6.06**
**Container Index**	4.04	1.15	0	0	6.32	2.22	9.79	0	**5.33**	**0.86**
**Breteau Index**	72.73	9.09	0	0	85.71	16.67	175	0	**87.5**	**6.06**
***Ae*. *aegypti* larvae (larvae/ vehicle)**	551 (50.09)	8 (0.73)	0	0	33 (4.71)	20 (3.33)	126 (15.75)	0	**710 (22.19)**	**28 (0.85)**
***Ae*. *aegypti* pupae (pupae/ vehicle)**	49 (4.45)	0	0	0	20 (2.9)	0	17 (2.13)	0	**86 (2.69)**	**0**

The data shown in the table below only includes independent instances of sampling for large (N = 32) and medium (N = 33) barges. Container (positive containers/ number inspected *100), Breteau (positive containers/ premises inspected*100), and Premise Indices (positive premises/ number inspected *100), were calculated using the presence of either larvae or pupae. Entomological indices were adapted for vehicle surveillance so that an individual vehicle was counted as a ‘premise’[18].

Interestingly, the proportion of vehicles positive for larvae or pupae was higher during sample dates with less precipitation, 30.8% for the May-August collections and 15.8% for the October-February collections. As with adult mosquitoes, the distribution of immature *Ae*. *aegypti* among barges was highly aggregated: 6.3% of barges (N = 2) produced 93.6% of larvae and 76.7% of pupae.

On average we found 16.7 wet containers per barge across all dates (SD = 10.3), with no difference between the low and high precipitation periods (17.9 containers/ vehicle SD = 12.8 vs 17.5 containers/ vehicle, SD = 9.0; U = 348, p>0.05). As with adult mosquitoes, immature *Ae*. *aegypti* were most likely to be found in cargo holds, accounting for 89.3% (N = 25) of positive habitats (Χ^2^ = 9.8, p<0.05). The remaining positive habitats were found on the first (7.1%, N = 2) and second floors (3.6%, N = 1) of barges. Difference in the number of larvae and pupae by floor approached significance, with a greater number of immature mosquitoes found in the cargo holds (larvae: Χ^2^ = 7.5, p = 0.06, pupae: Χ^2^ = 7.8, p = 0.05) (**Fig 4**).

The preferred habitats of larvae (85.9%) and pupae (76.7%) were puddles formed on the boat floor in cargo holds. Other containers produced immature mosquitoes, including tires (10.4% of larvae and 3.5% of pupae) and dishes (3.7% of larvae and 19.8% of pupae). The proportion of *Ae*. *aegypti* positive floor puddles, tires, and dishes was significantly greater in comparison with other container types (Fisher’s exact test p<0.05), but puddles in cargo holds were by far the most abundant habitat overall (N = 396). Other containers were relatively rare (**Table 4**). Despite thorough inspection, no mosquito eggs were found on barges, likely due to the extremely dark conditions in cargo holds.

**Table 4:** Proportion of positive containers by type—large barges.

	Large Barges	Medium Barges
	No. positive/ No. inspected (%)	No. positive/ No. inspected (%)
**Tires**	2/ 23 (8.70)	1/ 46 (2.17)
**Floor puddles**	25/ 396 (6.31)	0/ 91
**Dishes (plates, mugs, plate holders)**	1/ 20 (5)	0/ 13
**Other (tanks/ drums, plastic containers)**	0/80	0/ 78
**Trash (discarded items)**	0/ 6	1/5 (20)
**Total**	**28/ 525 (5.33)**	**2/ 233 (0.86)**

A container was considered to be positive if it contained *Ae*. *aegypti* at any immature stage (eggs, larvae, or pupae). On large barges significant differences were found in terms of floor puddles, dishes, and tires, and other container types (Fisher’s exact test p<0.05). On medium barges significant differences were detected between tires, trash, and other container types (Fisher’s exact test p<0.01).

Of the 14 barges that were sampled more than once, 13 were positive for *Ae*. *aegypti* immatures or adults during at least one sampling occasion. In general, barges that were initially infested tended to remain infested over time: only two boats were initially infested and later found to be uninfested.

### Medium Barges

On medium barges, 15 species of adult mosquitoes were found, with *Cx*. *quinquefasciatus* comprising 84.9% of all identified mosquitoes (**Table 1**, **S2 Table**). *Ae*. *aegypti* comprised 6.3% of mosquitoes, although overall abundance was significantly lower than for large barges (**Table 2**, N = 23 *Ae*. *aegypti* adults found among independent observations, U = 787.5, p<0.0001). There were no significant differences in adult *Ae*. *aegypti* abundance by date or by high vs. low precipitation period (Χ^2^ = 4.0, p> 0.05; U = 169, p>0.05, respectively).

Immature mosquitoes found on medium barges included *Ae*. *aegypti* and *Cx*. *declarator-mollis*. In comparison with large barges, immature indices on medium-sized barges were notably lower (**Table 3**). The proportion of boats positive for *Ae*. *aegypti* immatures was highest in August (Premise Index: 16.7), followed by February (Premise Index: 9.1). No immature mosquitoes were found on medium barges In May or in October. For all dates, about 0.9% of containers were positive (**Table 4**), and the types of containers infested differed significantly (tires, trash, and other container types, Fisher’s exact test p< 0.01).

In contrast to large barges, *Ae*. *aegypti* infestation status on medium barges was less consistent. Only three medium barges that were initially infested with *Ae*. *aegypti* remained infested in subsequent dates.

## Discussion

Few studies to date have actively monitored human transport vehicles for invasive species [19,25,34]. While *Ae*. *aegypti* has been previously documented on vehicles in Iquitos [18], we compared infestation across multiple vehicle types and across a full year capturing seasonal variation. We conclude that river boats are the most significant source *Ae*. *aegypti* regional spread in the Peruvian Amazon because 1) large and medium barges are frequently and heavily infested with mosquitoes, 2) the majority of towns in the Iquitos region are connected only by rivers, and 3) the spatial pattern of *Ae*. *aegypti* establishment in the region suggests a primarily riverine mode of spread [23]. Although buses traveled most frequently, their overall infestation rates were very low (no more than one mosquito per sampling event), and they traveled for much shorter distances

### *Ae*. *aegypti* and Dengue Invasion

Our results support the hypothesis that in the Peruvian Amazon aquatic transit is most important for the spread of *Ae*. *aegypti*. Although buses had a lower infestation rate in comparison with barges, terrestrial routes are important for trade and transportation in most of the rest of the world. Further study is needed to determine whether vehicle infestation rates are similar in other areas, and the degree to which terrestrial traffic contributes to *Ae*. *aegypti* invasion. Infestation rate, however, is only one component of propagule pressure, and a better measure of invasion risk would be the arrival of infested vehicles to new locations, as pointed out by Caton et al (2006). Infestation rate alone, therefore, may overestimate invasion risk, particularly when vehicle traffic is low, few mosquitoes are adults, or the male-female ratio is uneven [34].

Because there are no major highways in the Peruvian Amazon, regional transportation is predominantly aquatic: large barges frequently carry up to ~200 passengers to Iquitos from major population centers such as Pucallpa (approximately 200,000 inhabitants). It is therefore probable that infected individuals introduce dengue viruses to Iquitos by boat. Heavy mosquito infestation on boats could also lead to incipient virus transmission during travel. Trips can last several days (and in some cases weeks), leaving ample time for mosquitoes to take several blood meals. Stoddard et al (2014) described Amazon River levels as increasing during the first and third trimesters of the year (September-March), which are also periods of high dengue transmission. Since river traffic appears to be more intense during periods of intermediate river levels, we would expect more mosquito and dengue introductions during that time.

Dengue control strategies are very limited and focus on the reduction of mosquito infestations in urban areas where dengue cases have been detected: only rarely is control done preemptively. Results from this study, in conjunction with our previous findings [23], imply that *Ae*. *aegypti* mosquitoes move between Iquitos and surrounding towns. Population genetics studies could be used to characterize metapopulation structure of *Ae*. *aegypti* in the Iquitos region, and identify which routes (terrestrial or aquatic) are most relevant for gene flow. Evidence for fluid migration between subpopulations would indicate that rural settlements may serve as refuge sources for reinfestation or, given the limited vector control in such towns, potential sources of mosquitoes that are susceptible to insecticides.

### Knowledge Gaps about *Ae*. *aegypti* Infestation of Boats

It is not surprising that in the Peruvian Amazon *Ae*. *aegypti* colonization of barges is extremely common, given that these vehicles provide all of the mosquito’s lifecycle needs: a captive group of human hosts for blood meals, abundant oviposition sites, and dark, cool resting places for adults. There are two means by which mosquitoes might initially colonize a boat: flight of adults in search of oviposition sites [14] or humans unintentionally carrying infested containers aboard. It is unclear however, which of these mechanisms is most common and how seasonality might influence the colonization process. Mark-release-recapture experiments in empty port areas could be employed to compare the relative “attractiveness” of boats vs. houses in different seasons. Human-mediated dispersal could be measured through intensive longitudinal monitoring of artificial containers being loaded into vehicles. Of course, both mark-release-recapture experiments and longitudinal monitoring of artificial containers on boats would be labor-intensive. Indeed, despite inspecting 971 different containers on 34 different large barges, we were unable to locate a single mosquito egg.

**Fig 5:**
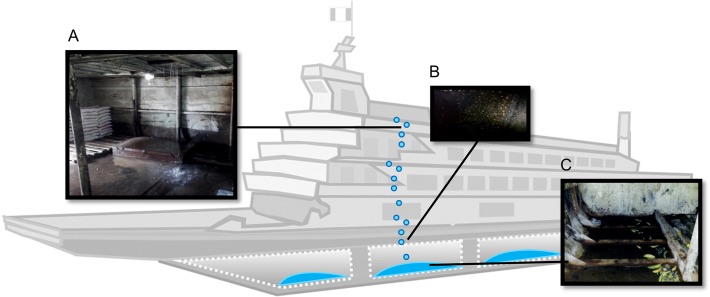
Formation of puddles in cargo holds. Barges are typically mopped whenever the boat is docked at either its origin or destination point. Water from rain and cleaning activities drips from upper most floors (A) through rust holes in the cargo hold roof (B). Water accumulates in the bottom of the cargo holds (C), and *Ae*. *aegypti* eggs are laid on the edges of the puddles, hatching when the puddles are refilled with water.

The most important seasonal change observed throughout the study was that collections from August (lower precipitation) showed a greater proportion of boats infested with immature *Ae*. *aegypti* (despite overall lower mosquito abundance). These patterns may be a result of *Ae*. *aegypti* searching more expansively for oviposition sites, due to scarcity of rain-filled wet containers in port areas during the periods of low precipitation [14]. Indeed, the number of wet containers on large barges did not differ between dates, possibly because the most common immature habitats (ground puddles in cargo holds) are primarily filled through cleaning activities and possibly through rainfall (**Fig 5**).

### Management of *Ae*. *aegypti* as an Invasive Species

Our findings indicate that periodic administration of larvicides alone is insufficient for mosquito control on boats. Despite administering temephos, we often found the same vehicle infested again some months later. Therefore, we propose an integrated approach that combines larvicide administration with habitat source reduction through improved boat construction, maintenance, and cleaning. Adulticides should also be employed during periods of high mosquito abundance. Though local law in Iquitos mandates that boats be sprayed periodically with insecticides, this is rarely done since the laws are not enforced and boat owners have no economic incentive to carry out mosquito control. Accordingly, we propose that governmental bodies invest in mosquito control activities through existing infrastructure for aquatic transit (such as port authorities). Both punitive policies (such as fines) and incentivizing policies (such as tax breaks) could be implemented to ensure individual cooperation with mosquito control activities. In some cases active surveillance and control of mosquito populations in airplanes and ports has been conducted to allow for early detection and rapid intervention of invasive species [19], although in resource-poor environments this unlikely to be a realistic solution. The aggregate distribution of adult and immature mosquitoes suggests that some boats may act as super-transporters of mosquitoes, just as individual hosts may act as super-spreaders of pathogens [35]. Taking this into account, vector control programs might target those vehicles producing the greatest amount of mosquitoes. In our collections, we observed that infested boats tended to be older, and were more likely to have rust holes that allow water to drip between floors and collect in cargo holds to form puddles.

### Disclaimer

The views expressed in this article are those of the authors and do not necessarily reflect the official policy or position of the Department of the Navy, Department of Defense, nor the U.S. Government. Author Helvio Astete is an employee of the U.S. Government. This work was prepared as part of his official duties. Title 17 U.S.C. §105 provides that ‘Copyright protection under this title is not available for any work of the United States Government.’ Title 17 U.S.C. §101 defines a U.S. Government work as a work prepared by a military service member or employee of the U.S. Government as part of that person’s official duties.

## Supporting Information

S1 Fig NOAA precipitation and rainfall data for Iquitos. A) Average monthly rainfall (cm) for 2009–2013 and B) Daily average, minimum, and maximum temperatures for 2009–2013. The symbol * on the graph indicates the months in which sampling took place.
(TIFF)

S1 Table Adult mosquitoes found on large barges by season. In some cases mosquito samples were damaged and could only be identified to genus or subgenus (denoted by spp.).
(DOCX)

S2 Table Adult mosquitoes found on medium barges by season. In some cases mosquito samples were damaged and could only be identified to genus or subgenus (denoted by spp.).
(DOCX)

S3 Table Adult mosquitoes found on buses by season. In some cases mosquito samples were damaged and could only be identified to genus or subgenus (denoted by spp.).
(DOCX)

S4 Table Adult mosquitoes found on speed boats by season. In some cases mosquito samples were damaged and could only be identified to genus or subgenus (denoted by spp.).
(DOCX)

S5 Table Adult mosquitoes found on water taxis by season. In some cases mosquito samples were damaged and could only be identified to genus or subgenus (denoted by spp.).
(DOCX)
