# Supplementary figures and images for: River Boats Contribute to the Regional Spread of the Dengue Vector Aedes aegypti in the Peruvian Amazon
Source: PLoS Negl Trop Dis. 2015 Apr 10;9(4):e0003648. doi: 10.1371/journal.pntd.0003648 (PMC4393238; doi:10.1371/journal.pntd.0003648)

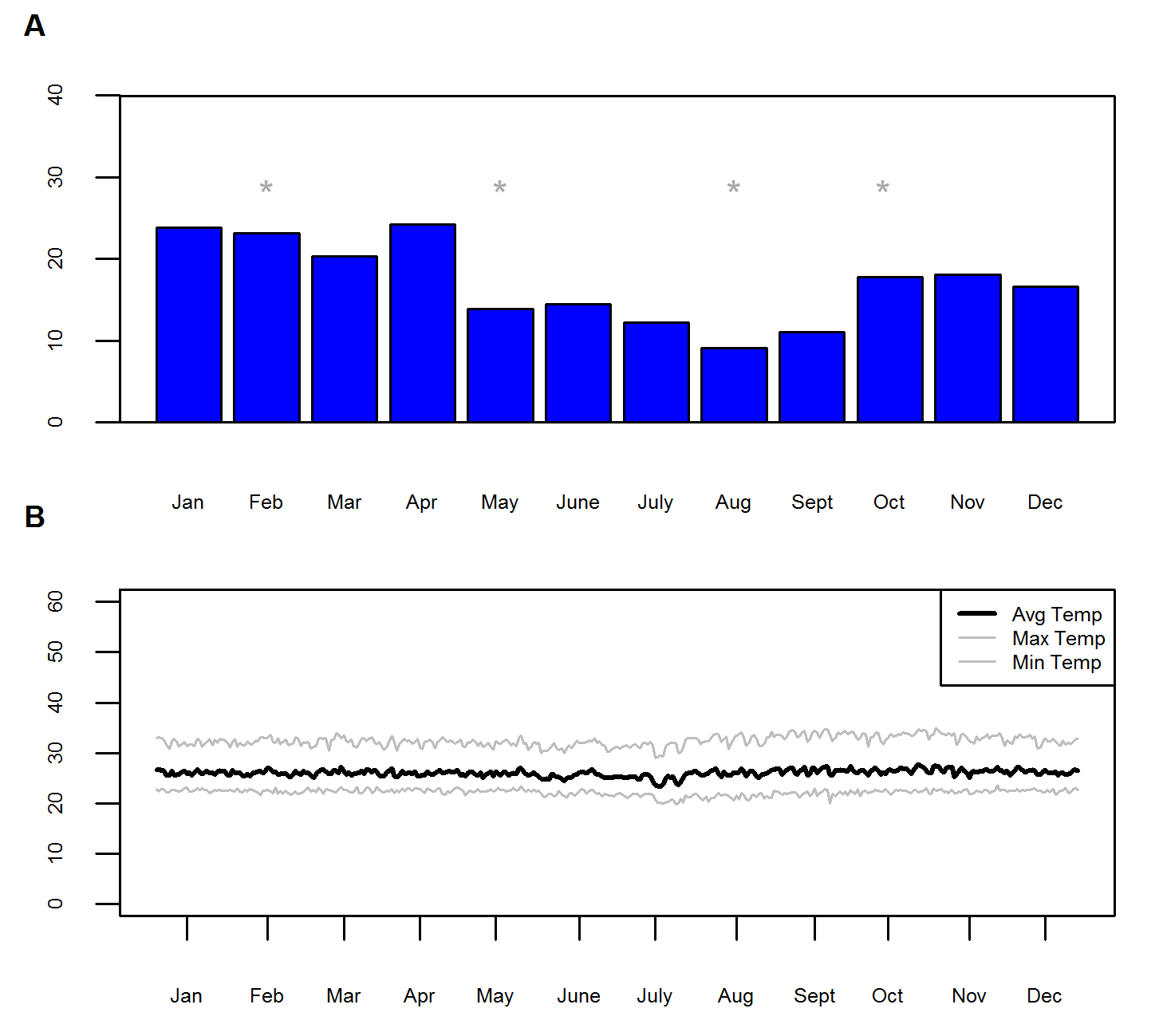

Supplement: S1 Fig — A) Average monthly rainfall (cm) for 2009–2013 and B) Daily average, minimum, and maximum temperatures for 2009–2013. The symbol * on the graph indicates the months in which sampling took place. (TIFF) [file pntd.0003648.s001.tiff]
