# Supplementary material for: River Boats Contribute to the Regional Spread of the Dengue Vector Aedes aegypti in the Peruvian Amazon
Source: PLoS Negl Trop Dis. 2015 Apr 10;9(4):e0003648. doi: 10.1371/journal.pntd.0003648 (PMC4393238; doi:10.1371/journal.pntd.0003648)
Supplement: S1 Table — In some cases mosquito samples were damaged and could only be identified to genus or subgenus (denoted by spp.). (DOCX) [file pntd.0003648.s002.docx]

**S1 Table. Adult mosquitoes found on large barges by season**. In some cases mosquito samples were damaged and could only be identified to genus or subgenus (denoted by spp.).

| **Genus** | **(Subgenus) species** | **All months** | **February** | **May** | **August** | **October** |
| --- | --- | --- | --- | --- | --- | --- |
| *Culex* |  |  |  |  |  |  |
|  | spp. | 3618 | 1299 | 657 | 352 | 1310 |
|  | (*Culex*) *quinquefasciatus* | 2409 | 697 | 510 | 219 | 983 |
|  | (*Culex*) *coronator* | 96 | 95 | 1 | 0 | 0 |
| *Aedes* |  |  |  |  |  |  |
|  | (*Stegomyia*) *aegypti* | 1110 | 89 | 144 | 52 | 825 |
| *Mansonia* |  |  |  |  |  |  |
|  | (*Mansonia*) *titillans* or *indubitans* | 13 | 2 | 5 | 5 | 1 |
|  | (*Mansonia*) *titillans* | 33 | 5 | 7 | 10 | 11 |
|  | (*Mansonia*) *indubitans* | 31 | 24 | 1 | 6 | 0 |
|  | (*Mansonia*) *humeralis* | 15 | 2 | 5 | 7 | 1 |
|  | (*Mansonia*) *amazonensis* | 1 | 0 | 1 | 0 | 0 |
| *Aedomyia* |  |  |  |  |  |  |
|  | (*Aedomyia*) *squamipennis* | 12 | 7 | 5 | 0 | 0 |
| *Coquillettidia* |  |  |  |  |  |  |
|  | (Rhynchotaenia) *venezuelensis* | 2 | 0 | 0 | 0 | 2 |
| *Limatus* |  |  |  |  |  |  |
|  | spp. | 1 | 1 | 0 | 0 | 0 |
|  | **Total** | **7469** | **2318** | **1354** | **654** | **3143** |
