# Supplementary material for: River Boats Contribute to the Regional Spread of the Dengue Vector Aedes aegypti in the Peruvian Amazon
Source: PLoS Negl Trop Dis. 2015 Apr 10;9(4):e0003648. doi: 10.1371/journal.pntd.0003648 (PMC4393238; doi:10.1371/journal.pntd.0003648)
Supplement: S2 Table — In some cases mosquito samples were damaged and could only be identified to genus or subgenus (denoted by spp.). (DOCX) [file pntd.0003648.s003.docx]

**S2 Table. Adult mosquitoes found on medium barges by season**. In some cases mosquito samples were damaged and could only be identified to genus or subgenus (denoted by spp.).

| **Genus** | **(Subgenus) species** | **All months** | **February** | **May** | **August** | **October** |
| --- | --- | --- | --- | --- | --- | --- |
| *Culex* |  |  |  |  |  |  |
|  | spp. | 1372 | 20 | 695 | 140 | 517 |
|  | (*Culex*) *quinquefasciatus* | 1060 | 9 | 579 | 65 | 407 |
|  | (*Culex*) *coronator* | 8 | 8 | 0 | 0 | **0** |
|  | *(Culex) declarator*-*mollis* | 30 | 5 | 25 | 0 | 0 |
|  | (*Phenacomyia*) corniger | 2 | 1 | 1 | 0 | 0 |
|  | (*Melanoconion*) spp. | 1 | 0 | 0 | 0 | 1 |
|  | *(Melanoconion) adamesi* | 8 | 8 | 0 | 0 | 0 |
|  | (*Melanoconion*) *spissipes* | 1 | 0 | 0 | 1 | 0 |
|  | (*Melanoconion*) *ocossa* | 1 | 0 | 0 | 0 | 1 |
|  | *(Aedinus*) *amazonensis* | 1 | 0 | 1 | 0 | 0 |
| *Aedes* |  |  |  |  |  |  |
|  | (*Stegomyia*) *aegypti* | 79 | 10 | 7 | 9 | 53 |
| *Mansonia* |  |  |  |  |  |  |
|  | (*Mansonia*) *titillans* or *indubitans* | 2 | 0 | 2 | 0 | 0 |
|  | (*Mansonia*) *titillans* | 20 | 5 | 3 | 5 | 7 |
|  | (*Mansonia*) *indubitans* | 19 | 1 | 10 | 8 | 0 |
|  | (*Mansonia*) *humeralis* | 9 | 1 | 3 | 2 | 3 |
| *Aedomyia* |  |  |  |  |  |  |
|  | (*Aedomyia*) *squamipennis* | 4 | 1 | 3 | 0 | 0 |
| *Coquillettidia* |  |  |  |  |  |  |
|  | spp. | 1 | 0 | 1 | 0 | 0 |
|  | (Rhynchotaenia) *venezuelensis* | 3 | 1 | 0 | 0 | 2 |
| *Ochlerotatus* |  |  |  |  |  |  |
|  | (*Protoculex*) *serratus* | 1 | 0 | 0 | 0 | 1 |
|  | **Total** | **2622** | **70** | **1330** | **230** | **992** |
