# Supplementary material for: River Boats Contribute to the Regional Spread of the Dengue Vector Aedes aegypti in the Peruvian Amazon
Source: PLoS Negl Trop Dis. 2015 Apr 10;9(4):e0003648. doi: 10.1371/journal.pntd.0003648 (PMC4393238; doi:10.1371/journal.pntd.0003648)
Supplement: S3 Table — In some cases mosquito samples were damaged and could only be identified to genus or subgenus (denoted by spp.). (DOCX) [file pntd.0003648.s004.docx]

**S3 Table. Adult mosquitoes found on buses by season**. In some cases mosquito samples were damaged and could only be identified to genus or subgenus (denoted by spp.).

| **Genus** | **(Subgenus) species** | **All months** | **February** | **May** | **August** | **October** |
| --- | --- | --- | --- | --- | --- | --- |
| *Culex* |  |  |  |  |  |  |
|  | spp. | 8 | 6 | 2 | 0 | 0 |
|  | (*Culex*) spp. | 3 | 0 | 0 | 3 | 0 |
|  | (*Culex*) *quinquefasciatus* | 4 | 1 | 2 | 0 | 1 |
| *Aedes* |  |  |  |  |  |  |
|  | (*Stegomyia*) *aegypti* | 7 | 3 | 3 | 0 | 1 |
| *Anopheles* |  |  |  |  |  |  |
|  | spp. | 1 | 1 | 0 | 0 | 0 |
|  | **Total** | **23** | **11** | **7** | **3** | **2** |
