# Supplementary material for: River Boats Contribute to the Regional Spread of the Dengue Vector Aedes aegypti in the Peruvian Amazon
Source: PLoS Negl Trop Dis. 2015 Apr 10;9(4):e0003648. doi: 10.1371/journal.pntd.0003648 (PMC4393238; doi:10.1371/journal.pntd.0003648)
Supplement: S5 Table — In some cases mosquito samples were damaged and could only be identified to genus or subgenus (denoted by spp.). (DOCX) [file pntd.0003648.s006.docx]

**S5 Table. Adult mosquitoes found on water taxis by season**. In some cases mosquito samples were damaged and could only be identified to genus or subgenus (denoted by spp.).

| **Genus** | **(Subgenus) species** | **All months** | **February** | **May** | **August** | **October** |
| --- | --- | --- | --- | --- | --- | --- |
| *Culex* |  |  |  |  |  |  |
|  | *(Melanoconion)* spp. | 1 | 0 | 1 | 0 | 0 |
|  | **Total** | **1** | **0** | **1** | **0** | **0** |
